# Prophylactic effect of flavanol rich preparation metabolites in promoting resilience to a mouse model of social stress

**DOI:** 10.1038/s41398-020-0859-x

**Published:** 2020-06-09

**Authors:** Jun Wang, Danyue Zhao, Simoni Tiano, Adelaida Esteban-Fernández, Bo Yuan, Chad Smith, Justin Brathwaite, Zahra Jlayer, Qingli Wu, James E. Simon, Kyle J. Trageser, Giulio M. Pasinetti

**Affiliations:** 1 Department of Neurology, Icahn School of Medicine at Mount Sinai, New York, NY 10029 USA; 2 Geriatric Research, Education and Clinical Center, James J. Peters Veterans Affairs Medical Center, Bronx, New York, NY 10468 USA; 3 New Use Agriculture & Natural Plant Products Program, Department of Plant Biology, School of Environmental and Biological Sciences, Rutgers University, New Brunswick, NJ 08901 USA

**Keywords:** Molecular neuroscience, Psychology

## Abstract

Major depressive disorder (MDD) is a leading cause of disability, and there is an urgent need for new therapeutics. Stress-mediated induction of pro-inflammation in the periphery contributes to depression-like behaviors both in humans and in experimental models. Inflammatory cytokine interleukin-6 (IL-6) has emerged as a potential therapeutic target. Our studies demonstrated that metabolism of flavanol rich cocoa preparation (FRP) led to the accumulation of select phenolic acids that may contribute to its anti-inflammatory activity. Using a repeated social defeat stress (RSDS) model of depression, we showed that oral administration of FRP attenuates susceptibility to RSDS-mediated depression, supporting the further development of FRP as a novel therapeutic for the treatment of stress disorders and anxiety in humans.

## Introduction

Major depressive disorder (MDD) takes an enormous toll on daily life and normal functioning. Currently, available treatments for depression target neurochemical or neurobiological mechanisms identified retrospectively following the discovery of the drug’s initial antidepressant efficacy^1^. As conventional pharmacological treatments are estimated to produce temporary remission in <50% of patients and are often accompanied by a wide range of adverse events^2,3^, there is a need for a wider spectrum of novel therapeutics to target newly discovered underlying disease mechanisms.

MDD is a multifaceted disease and the etiology is not completely understood. The contribution of the central nervous system (CNS) in the pathophysiology of MDD has been extensively studied^4–7^. In recent decades, peripheral inflammation has been recognized as one of the emerging mechanisms of MDD and potential therapeutic targets^8–13^. Although many neuroimmune factors have been implicated in depressive disorders, studies in humans suggest that elevation of peripheral IL-6 is one factor that is most consistently observed^14–16^. Moreover, in a repeated social defeat stress (RSDS) mouse model of depression, it was reported that mice show individual differences in IL-6 production in response to RSDS, and that this intrinsic immune characteristic predicts susceptibility versus resilience to RSDS. Sequestering and neutralizing peripheral IL-6 using monoclonal antibodies significantly promotes resilience to RSDS-induced depression phenotypes^17^. While the mechanisms by which peripheral IL-6 may modulate depression phenotypes is currently under intense investigation, evidence suggest that in both human and in rodent models of depression, chronic social stress alters blood vessel ultrastructure, and in combination with stress-induced peripheral inflammation, increases blood brain barrier (BBB) leakiness that allows the infiltration of inflammatory molecules into the brain^18^. Once IL-6 crosses the BBB, it may alter neuroplasticity and modulate brain activity by acting directly on neurons, or indirectly through modulation of microglia and/or other CNS immune cells^19^. These studies suggest that modulation of IL-6 and associated immune signaling pathways may provide a novel therapeutic strategy to prevent and/or treat depression.

Cocoa is the dried seed of *Theobroma cacao*, and is a rich source of polyphenols, particularly epicatechins^20,21^. Previously, we found an epicatechin-rich grape polyphenol extract that was highly effective in protecting against the onset and/or progression of multiple, diverse neurological, psychological, and metabolic disorders in animal models, mainly due to its bioactivities in modulating synaptic function^22–28^. Published evidence suggests cocoa consumption (in the form of chocolate) may improve cognitive function^29^ and reduce fatigue^30^. Clinical observation also revealed that cocoa may beneficially modulate mood^29^. The biological activities/properties of cocoa are modulated by the bioavailability of its bioactive constituents which include polyphenols^20,21^. Cocoa flavanols have been shown to possess many biological activities including strong anti-inflammatory activities in vitro. Cocoa flavanols are capable of modulating TNF-α and IL-1β in peripheral blood mononuclear cells (PBMCs)^31,32^. Cocoa proanthocyanidin monomers and dimmers can also inhibit nuclear factor кB (NF-кB) activation^33^. Based on these observations and the emerging significance of inflammation in the pathogenesis of MDD, the current study tests the efficacy of a specific flavanol rich cocoa preparation (FRP) in attenuating depression in the repeated social defeat stress (RSDS) mouse model of depression.

## Materials and methods

Commercially available FRP CocoaVia® was purchased from Cocoavia.com (Lot number 144533, MARS International). The cocoa extract was chemically profiled for polyphenols (see below) and archived in compliance with NCCIH Product Integrity guidelines. Phenolic standards including gallic acid (GA), caffeic acid (CA), *trans*-*p*-coumaric acid (*p*-CA), catechin ((+)-C), epicatechin ((−)-EC), dihydrocoumaric acid (diHCA), 3-(3,4-dihydroxyphenyl)propionic acid (3,4-diHPPA), 3,4-dihydroxybenzoic acid (3,4-diHBA), hippuric acid (HA), homovanillic acid (HVA), 3-hydroxybenzoic acid (3-HBA), 4-hydroxybenzoic acid (4-HBA), 3-hydroxyphenylacetic acid (3-HPAA), 3-(3-hydroxyphenyl)propionic acid (3-HPPA), vanillic acid (VA), and *trans*-cinnamic acid-d_7_ (internal standard) were purchased from Sigma-Aldrich (St. Louis, MO, USA); 3,4-dihydroxyphenylacetic acid (3,4-diHPAA) and ferulic acid (FA) were from ChromaDex Inc. (Irvine, CA); 5-(4-hydroxyphenyl)valeric acid (4-HPVA) was from Alfa Aesar (UK). Acids and solvents (all HPLC Grade) including glacial acetic acid (AA), formic acid (FA), acetonitrile (ACN), methanol (MeOH) and ethyl acetate were obtained from Fisher Scientific (Pittsburgh, PA, USA), and Pierce™ LC-MS water from Thermo Fisher Scientific (Waltham, MA, USA).

### Animals

All C57BL/6 mice were purchased from the Jackson Laboratory (Bar Harbor, ME). Retired breeder CD-1 mice were purchased from Charles River Laboratory (Wilmington, MA). All animals had access to regular chow ad lib and were maintained on a 12:12-h light/dark cycle with lights on at 07:00 h in a temperature-controlled (20 ± 2 °C) vivarium and all procedures were approved by the Mount Sinai Institutional Animal Care and Use Committee.

### Treatment

For the dose-finding study, 8-week-old male C57BL/6 mice were randomly allocated into experimental groups and fed a polyphenol-free diet for 10 days and then treated with vehicle, 8, 40, 200, and 500 mg/kg body weight (BW)/day FRP, delivered through their drinking water for 2 weeks. Mice were then challenged with intraperitoneal (i.p.) injection of 0.4 mg/kg-BW lipopolysaccharide (LPS) and plasma levels of IL-6 were measured 6 h post injection. For the bioavailability study, 6-week-old male C57BL/6 mice were fed with polyphenol-free diet for 10 days and then treated with a vehicle, 40 mg/kg-BW/day or 200 mg/kg-BW/day FRP for 2 weeks before sacrificing. For the in vivo efficacy study, 6-week-old male C57BL/6 mice were fed a polyphenol-free diet for 10 days and the mice were then randomly grouped into two: one group received regular drinking water and the other group received FRP-adulterated water (40 mg/kg-BW/day), starting 2 weeks prior to RSDS and throughout RSDS and social interaction (SI) testing.

### Quantitative analysis of the flavanol rich cocoa preparation

For LC-DAD-MS analysis, a Hewlett Packard Agilent 1100 Series LC/MS (Agilent Technologies, Waldbronn, Germany) equipped with autosampler, quaternary pump system, DAD detector, degasser, MSD trap with an electrospray ion (ESI) source was employed. Data acquisition and processing was achieved using HP ChemStation, Bruker Daltonics and DataAnalysis software (Agilent, ver 4.1). Chromatographic separation was performed on a Polaris amide-C18 column, 250 × 4.6 mm, 5um (Varian Inc.) with a binary mobile phase containing solvent A (0.1% FA in water) and B (0.1% FA in ACN). Elution gradient started at 10% B and linearly increased to 20% in 20 min; during 20–30 min, linearly increase to 30% B; during 30–40 min, isocratic elution at 30% B with a flow rate of 1.0 mL/min. The UV detector was set to monitor at 254 (for hippuric acid), 280 (for phenolic acid & flavanols), 370 (for flavonols), and 520 (for anthocyanidins) nm. Mass spectral data acquisition was achieved under positive polarity (ESI+) with a needle voltage at 3.5 kV and scanned from *m/z* 100 to 1200. Nitrogen was used as dry gas at a flow rate of 12 L/min and capillary temperature was at 350 **°**C. Nitrogen was used as nebulizer gas at 60 psi, and helium as collision gas.

### Plasma bioavailability

Eight-week-old male C57BL/6 mice were fed a polyphenol-free diet for 10 days and then treated with a vehicle, 40 mg/kg-BW/day or 200 mg/kg-BW/day FRP for 2 weeks. On the day of sacrifice, mice were gavaged with a daily dose of the FRP and blood samples were collected one hour (for phase II polyphenol conjugates detection) or 6 h (for phenolic acids detection) following the gavage. Plasma was collected by centrifugation at 1500×*g* for 10 min and formic acid was added to the plasma to a final concentration of 0.2%. Samples were snap frozen and stored at −80 °C until further analysis.

#### Analysis of Phase II metabolites of polyphenol conjugates

##### Sample preparation

Plasma samples were previously stored in −80 °C freezer and transferred to −20 °C 24 h prior to analysis, thawed on ice, and conditioned to room temperature before processing. A thawed aliquot of plasma (100 µL) was added to 500 µL methanol containing 2% acetic acid and vortexed for 30 s. The sample was allowed to stand still for 5 min for complete protein precipitation, followed by centrifugation at 16,000×*g* for 10 min. The precipitate was re-mixed with 200 µL of acidified methanol and extracted one more time. The pooled supernatant was then transferred to a glass vial and dried under a gentle stream of nitrogen. The residues were then reconstituted in 200 µL 20% acetonitrile in water containing 0.1% formic acid. The reconstituted sample was centrifuged at 16 000×*g* for 10 min and 10 µL of the supernatant was injected into the LC-MS/MS system.

##### Phase II metabolites analysis

Phase II metabolites from plasma samples were analyzed using an Agilent 1290 Infinity II UPLC (Agilent Technology, Atlanta, GA, USA) system interfaced with an Agilent 6400 Triple Quadrupole Mass Spectrometer (LC-QqQ/MS) with an ESI source. A Waters ACQUITY UPLC BEH C18 Column (2.1 × 50 mm with 1.7 μm particle size) was used with a thermostat set at 30 °C. The binary mobile phase consisted of 0.1% formic acid in water (solvent A) and 0.1% formic acid in acetonitrile (solvent B). The flow rate was set to 0.4 mL/min. The gradient conditions used were 2% B at 0 min, 5% B at 6 min, 25% B at 10 min, 95% B at 12 min, and back to 2% B at 13 min with 2 min post-run equilibration. The MS was operated with positive polarity under multiple reaction monitoring (MRM) mode. The MRM transition for (+)-catechin (C) and (−)-epicatechin (EC) was 291 → 139, the transition for C 3′-O-glucuronide (C-glucur) and EC 3′-O-glucuronide (EC-glucur) was 467 → 291, and the transition for Methyl O-C-glucuronide (Me-C-glucur) and Methyl O-EC-glucuronide (Me-EC-glucur) was 481 → 305. The fragment voltage used was set at 106 V, the collision energy at 12 eV and the cell accelerator voltage at 4 V. The ESI conditions were set with the nebulizer pressure at 30 psi, the capillary voltage at 3500 V and the nozzle voltage at 1000 V, the drying gas temperature at 350 °C with a flow rate of 12 L/min, and the sheath gas temperature at 350 °C with a flow rate of 12 L/min. Glucuronides of C and EC was estimated using a calibration curve constructed with an authentic quercetin 3-O-glucuronide (Quer-gluc) standard and corrected by a molecular weight ratio (metabolite/Quer-gluc ratio). Methylated C/EC was quantified using the same calibration curve for C/EC.

#### Phenolic acid metabolite analysis

##### Sample preparation

Plasma samples were previously stored at −80 °C and transferred to −20 °C 24 h prior to analysis. Immediately before processing, samples were thawed on ice and then conditioned to room temperature. An internal standard (I.S.), *trans*-cinnamic acid (d_7_) (2 µg/mL) was mixed with 0.4 M NaH_2_PO_4_ buffer (pH 5.0) and a 300-µL aliquot containing 5 µL of I.S. was added to 100 µL of thawed plasma. The mixture was then incubated with 300 of NaH_2_PO_4_ buffer and 50 µL of the solution of β-glucuronidase (2000 U) in contamination with sulfatase (diluted in NaH_2_PO_4_ buffer). For plasma samples with volume less than 100 µL, enzymatic digestion was carried out directly in the original Eppendorf tube as aforementioned. All samples were incubated at 37 °C for 45 min after purging with nitrogen. Enzymatic reaction was stopped by adding 500 μL of ethyl acetate. The mixture was vortexed vigorously for 1 min, followed by centrifugation at 3000×*g* for 5 min. The upper organic phase was transferred to a 1-dram glass vial. After two more extractions with ethyl acetate (500 μL), the pooled supernatant was mixed with 10 μL of 2% ascorbic acid in methanol and dried under a gentle stream of nitrogen. The residue was reconstituted in 100 μL of 45% methanol containing 0.1% formic acid and centrifuged at 16,000×*g* for 10 min.

##### Phenolic acids analysis

The analyses of C, EC, and phenolic acid metabolites (PAMs) were carried out on an Agilent 1290 Infinity II UPLC system interfaced with an Agilent 6470 Triple Quadrupole Mass Spectrometer with an ESI source (Agilent Technology, Palo Alto, CA, USA). For each sample extract, 5 μL was injected into an UPLC-QqQ/MS system for analysis using the method developed under dynamic multiple reaction monitoring (dMRM) mode.

### Assessment of IL-6 expression in vitro using periphery blood mononuclear cells (PBMCs)

PBMCs from 2-month-old mice were isolated using the Ficoll-Paque density-gradient centrifugation method. Specifically, whole blood was mixed with complete RPMI media and laid over the Ficoll-Paque Plus (GE Healthcare, Sweden), centrifuged at 2200 RPM for 15 min. The buffy coat containing PBMCs were isolated, washed once with BEP (0.5% BSA, 2 mM EDTA in PBS) and plated out in a 24-well plate at 5×10^5^/well in culture medium RPMI-1640 (Sigma) supplemented with 20% horse serum, 10% FBS, 2.05 mM l-glutamine, 25 mM Hepes, and 100 U/ml penicillin/streptomycin. PBMCs were treated with phenolic acids for 16 h and challenged with 7.5 µg/ml LPS. Supernatant was collected by centrifugation following 16 h of LPS treatment and the levels of cytokines were measured using the Multiplex MAP mouse cytokine/chemokine Panel (EMD Millipore) following the manufacturer’s instruction.

### Behavioral testing

#### RSDS

RSDS was performed as previously described^34,35^. CD-1 mice were screened for aggressive characteristics prior to the start of social defeat experiments based on previously described criteria^35^, and housed in the social defeat cage (26.7w × 48.3d × 15.2 h cm; Allentown Inc) 24 h prior to the start of defeats on one side of a clear, perforated Plexiglass divider (0.6 × 45.7 × 15.2 cm; Nationwide Plastics). Briefly, mice subjected to RSDS were exposed to a novel CD-1 aggressor mouse for 10 min once per day, over 10 consecutive days. Following the 10-min interaction, the experimental mice were removed to the opposite side of the social defeat cage and sensory contact during the following 24-h period was allowed. Mice were returned to a single house following the last defeat and before the social avoidance test.

#### Social interaction test (social avoidance test)

Social interaction (SI) testing was performed as previously described^35^. All SI testings were performed under red-light conditions. Mice were placed in a novel interaction open-field arena custom-crafted from opaque Plexiglas (42 × 42 × 42 cm; Nationwide Plastics) with a small animal cage placed at one end. Their movements were then automatically monitored and recorded (Ethovision 3.0; Noldus Information Technology) for 2.5 min in the absence (target absent phase) of a novel CD-1 mouse. This phase is used to determine baseline exploratory behavior. We then immediately measured 2.5 min of exploratory behavior in the presence of a caged CD-1 mouse (target present phase), again recording the total distance traveled and the duration of time spent in the interaction and corner zones. SI behavior was then calculated as total time spent in each zone, or as a ratio of the time spent in the interaction with the target present divided by the time spent in the interaction zone with the target absent. All mice with a ratio above 1.0 were classified as resilient whereas below 1.0 were classified as susceptible.

### Overall statistics

All values are expressed as mean and standard error of the mean (s.e.m.). Unpaired two-tailed Student’s *t*-tests with Welch’s correction were used. In all studies, outliers (2 SD from the mean) were excluded and the null hypothesis was rejected at the 0.05 level. All statistical analyses were performed using Prism Stat program (GraphPad Software, Inc.).

## Results

### Flavanol identification and quantification

**Fig. 1:**
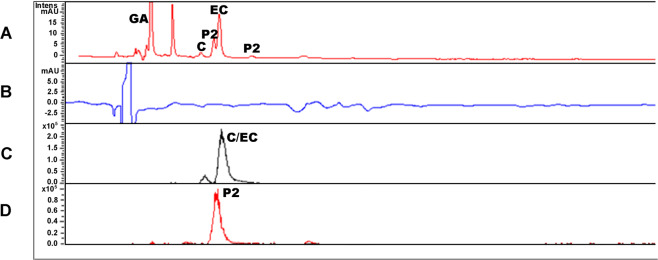
Chromatograms for FRP. **a** 280 nm chromatogram showing phenolic acids and PACs (GA = gallic acid, C = catechin, EC = epicatechin, P2 = proanthocyanidin dimer); **b** 370 nm chromatogram showing lack of flavanols; **c** Extracted ion chromatogram for catechin/epicatechin, *m/z* = 291, [M+H]^+^; **d** Extracted ion chromatogram for proanthocyanidin (PAC) dimers (P2), *m/z* = 579, [M+H]^+^.

Cocoa contains a high content of the flavonoids (−)-epicatechin, (+)-catechin and their dimers, proanthocyanidin B1 and B2, and the composition largely depends on the type of cocoa beans and the extraction methods. We measured the content of flavanols in the FRP under positive ionization mode with UV detection. The representative UV chromatograms (280 nm) and MS chromatograms of FRP polyphenols are illustrated in Fig. 1. On the basis of UV and MS spectral data and by comparison to the retention time of the authentic standards, we were able to detect catechin, epicatechin, and proanthocyanidin (PAC) dimers. The FRP contains approximately 1.92% of catechin, 7.97% of epicatechin, 6.97% of PAC dimers and 5.62% of gallic acid. We did not detect a measurable amount of flavanols, galloylated PAC dimers/trimers or PAC trimers/tetramers (Table 1).

**Table 1:** Quantitative analysis of FRP.

Compound	mg/g (%)
Catechin	19.2 ± 0.176 (1.92)
Epicatechin	79.7 ± 0.315 (7.97)
P2 isomers	69.7 ± 0.608 (6.97)
Gallic acid	56.2 ± 0.658 (5.62)

Chemical composition of FRP was quantified using HPLC/UV/MS: P2 = proanthocyanidin dimer.

### In vivo dose finding studies for FRP

**Fig. 2:**
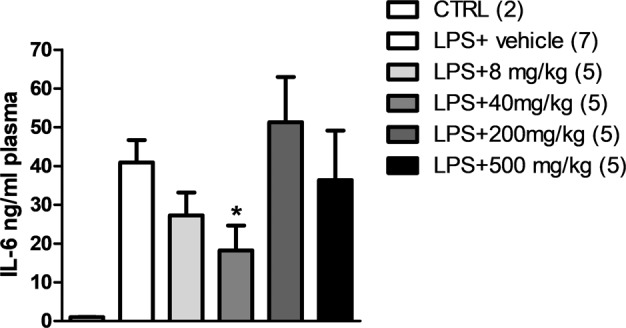
Dose finding study in C57BL6 mice. IL-6 levels in plasma six hours post LPS challenge in mice treated with various doses of FRP for 2 weeks (One-way ANOVA *F*5,20 = 2.82, *P* = 0.0385, *n* = 2 for CTRL). All bar graphs represent mean ± s.e.m., *P < 0.05.

In this study, we chose to target peripheral inflammation. We initiated dose response studies in mice to determine testing dosages for preclinical efficacy as a potential intervention for stress-induced depression. C57BL/6 wild-type mice were treated with FRP (doses ranging from 8 mg to 500 mg/kg-BW/day) for 2 weeks to simulate long-term administration. Mice were then challenged with intraperitoneal (i.p.) injection of 0.4 mg/kg LPS and plasma levels of IL-6 were measured 6 h post-injection. We found that pretreatment with lower doses of FRP led to a dose-dependent suppression of IL-6 production in plasma and that the group treated with 40 mg/kg-BW/day showed significant reduction compared to the vehicle-treated group. However, treatment with 200 and 500 mg/kg-BW/day did not show any effect in reducing LPS-mediated peripheral increase of IL-6 (Fig. 2).

### Plasma bioavailability studies

**Fig. 3:**
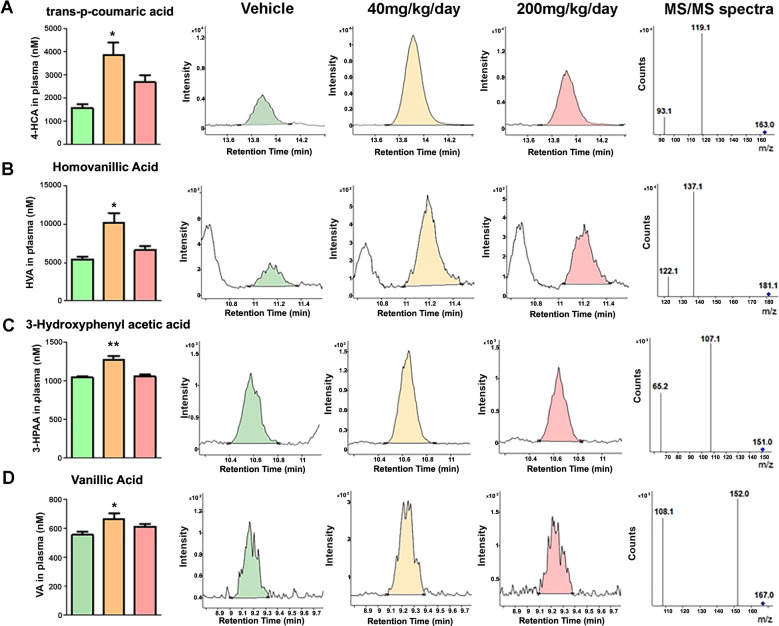
Selective increase of phenolic acids in plasma from mice treated with 40 mg/kg/day FRP compared to 200 mg/kg/day FRP. Plasma level of select phenolic acids were measured 6 h following the last dosing in mice treated with 40 mg/kg/day or 200 mg/kg/day for 2 weeks. **a** Plasma levels and representative mass spectra, MS/MS spectra of 4-HCA (One-way ANOVA, *F*_2,17_ = 5.75, *P* = 0.014). **b** Plasma levels and representative mass spectra, MS/MS spectra of HVA (One-way ANOVA, *F*_2,17_ = 6.08, *P* = 0.010). **c** Plasma levels and representative mass spectra, MS/MS spectra of 3-HPAA (One-way ANOVA, *F*_2,17_ = 8.85, *P* = 0.0023). **d** Plasma levels and representative mass spectra, MS/MS spectra of VA (One-way ANOVA, *F*_2,17_ = 3.60, *P* = 0.05). Bar graphs represent mean ± s.e.m., *P < 0.05. **P < 0.01.

Based on the observation that 40 mg/kg-BW/day significantly reduced LPS-induced IL-6 production in plasma while neither 200 nor 500 mg/kg-BW/day had effects, we reasoned that the differences may be due to the cumulative effect of polyphenol metabolites in the plasma following oral administration. To investigate qualitatively as well quantitatively the differences between 40 and 200 mg/kg-BW/day dosage, we initiated a bioavailability study. The majority of orally consumed dietary polyphenols, including those in FRP, are extensively metabolized during gastrointestinal absorption and/or post-absorptive xenobiotic metabolism, converting them into phase II polyphenol conjugate metabolites. In addition, polyphenols can also be metabolized by intestinal bacteria into phenolic acids^36,37^. Thus, orally consumed polyphenols are typically bioavailable in vivo as phenolic metabolites (polyphenol conjugate metabolites and phenolic acids). Previously we have shown that polyphenols from a flavanol-rich grape seed polyphenol extract (GSPE) were metabolized to phase II polyphenol conjugates fairly rapidly and peaked about one hour following oral gavaging^26^. On the other hand, it took roughly 6 h for the microbiome-derived phenolic acids to peak^38^. One group of mice was sacrificed 60 min following the final dose of gavaging and plasma was evaluated for phase II conjugates. Not surprisingly, we found a dose-dependent increase of phase II polyphenol metabolites in the forms of glucuronidated or methylated and glucuronidated catechin/epicatechin (Table 2). The second group of mice was sacrificed 6 h after the final dosing and plasma was evaluated for phenolic acid metabolites (PAMs). We found a dose-dependent increase of gallic acid, 3,4-dihydroxybenoic acid, 5-(4-hydroxyphenyl) valeric acid, and hippuric acid (Table 3). Interestingly, we also found a significant increase of *trans*-ρ-coumaric acid (4-HCA), homovanillic acid (HVA), 3-hydroxyphenylacetic acid (3-HPAA), and vanillic acid (VA) in the 40 mg/kg-BW/day compared to the vehicle control group and the 200 mg/kg-BW/day group (Fig. 3). We did not find significant changes in the amount of 3-hydroxybenzoic acid, dihydrocoumaric acid, 4-hydroxybenzoic acid, 3-(3-hydroxyphenyl) propionic acid, ferulic acid, or 3,4-dihydroxyphenylacetic acid (Table 3), suggesting these phenolic acids are very unlikely to be derived from FRP consumption, rather endogenously available. It is also possible that the production and excretion of these phenolic acid metabolites reach a dynamic equilibrium in the body and FRP consumption does not significantly affect their levels.

**Table 2:** Measurements of phase II polyphenol conjugates in plasma following FRP treatment.

Phase II	Vehicle CTRL		40 mg/kg/day		200 mg/kg/day	
Polyphenol	Average (nM)	St dev	Average (nM)	St dev	Average (nM)	St dev
Me-C-Gluc	N.D.		0.70	0.23	1.81	1.09
Me-C/EC-Gluc*	N.D.		1.39	0.81	3.28	1.69
Me-EC-Gluc	N.D.		1.66	0.92	4.42	2.55
C-Gluc	N.D.		2.29	2.88	11.37	3.06
EC-Gluc	N.D.		3.47	3.06	16.97	4.87
C	N.D.		5.34	2.28	15.40	3.81
EC	N.D.		10.27	7.41	35.36	12.69

Plasma levels of 3′-O-methyl-catechin-5-O-β-glucuronide (Me-C-Gluc), methyl-catechin/epicatechin-glucuronide (Me-C/EC-Gluc), 3′-O-methyl-epicatechin-5-O-β-glucuronide (Me-EC-Gluc), catechin-glucuronide (C-Gluc), epicatechin-glucuronide (EC-Gluc), catechin (C), and epicatechin (EC) measured 1 h following the last dosing in mice treated with 40 mg/kg/day or 200 mg/kg/day of FRP for 2 weeks, *n* = 5 for vehicle control, *n* = 7 for treatment group.

**Table 3:** Measurements of phenolic acids in plasma following FRP treatment.

Phenolic acids	Vehicle CTRL		40 mg/kg/day		200 mg/kg/day	
	Average (nM)	St dev	Average (nM)	St dev	Average (nM)	St dev
GA^a^	38.9	12.4	72.3	23.5	126.4	31.8
3,4-diHBA^a^	14.0	11.7	185.9	47.3	247.8	73.4
3,4-diHPPA	459.2	59.2	566.6	139.0	525.8	133.8
4-HBA	4174.6	191.6	4335.2	253.7	4152.1	184.9
HA^a^	10.5	14.4	125.7	65.0	641.7	566.6
VA^b^	555.2	63.1	667.4	99.8	614.9	40.8
3-HBA	579.4	10.3	578.7	12.4	587.8	12.0
3-HPPA^b^	1046.8	32.8	1228.7	125.1	1039.8	51.4
HVA^b^	5161.8	470.9	8952.9	1950.4	7045.7	1048.6
diHCA	205123.3	5945.6	195755.1	6280.6	174706.9	15465.6
4-HCA^b^	1535.3	224.6	3456.7	1119.1	2670.5	738.4
3-HPPA	468.5	4.6	505.0	60.8	513.1	18.8
FA	308.8	9.5	360.1	40.3	352.8	21.9
4-HPVA	0.0	0.0	Trace	0.0	31.4	11.4

Plasma levels phenolic acids measured 6 h following the last dosing in mice treated with vehicle, 40 mg/kg/day or 200 mg/kg/day of FRP for 2 weeks.

^a^Phenolic content significantly higher in the 200 mg/kg/day group compared to 40 mg/kg/day group.

^b^Phenolic content significantly higher in the 40 mg/kg/day group compared to 200 mg/kg/day group, *n* = 5 for vehicle control, *n* = 7–8 for treatment group.

### In vitro testing of the anti-inflammatory activity of 4-HCA, HVA, 3-HPAA, and VA

**Fig. 4:**
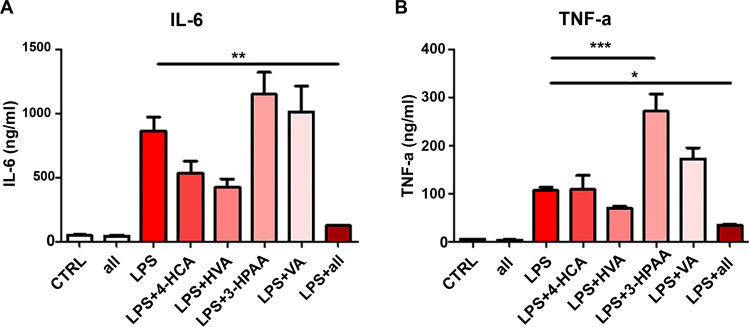
In vitro testing of phenolic acids in attenuating LPS-induced production of inflammatory cytokines. Mouse PBMCs were treated with either individual phenolic acid or the combination of all 4 phenolic acids that found to be significantly higher in the 40mg/kg/day group for 16h and stimulated with LPS for 16h. Cytokine content in the supernatant was measured by multiplex ELISA. **a** The level of IL-6 (One-way ANOVA, *F*_7,31_ = 15.95, *P* < 0.0001, *n* = 4 per culture conditions) and **b** the level of TNF-α (One-way ANOVA, *F*_7,31_ = 28.46, *P* < 0.0001, *n* = 4 per culture conditions). Bar graphs represent mean ± s.e.m., *P < 0.05, **P < 0.01, ***P < 0.001.

The bioavailability data showed that the level of 4-HCA, HVA, 3-HPAA, and VA were significantly higher in the 40-mg/kg-BW/day group compared to the vehicle-treated group, while 200 mg/kg-BW/day had similar amounts as the vehicle-treated group. The dose finding studies demonstrated that 40 mg/kg/day FRP can significantly reduce the LPS-mediated peripheral IL-6 induction, while 200 mg/kg/day treatment had no effect, with similar levels of IL-6 relative to the vehicle treatment. We hypothesized that these phenolic acids might be, in part, responsible for the attenuation of LPS-induced IL-6. To test this hypothesis, we treated peripheral blood mononuclear cells (PBMCs) isolated from wild-type C57BL6 mice with these four compounds either alone or in combination for 16 h. The concentrations we used were the same concentrations as identified in the plasma following 40 mg/kg/day treatment: 4 µM 4-HCA, 10 µM HVA, 1.25 µM 3-HPAA, and 0.7 µM VA. Following the treatment, PBMCs culture were challenged with 1 µg/ml LPS for 16 h and cytokines were measured using a Multiplex MAP mouse cytokine/chemokine panel. Interestingly, we found that when pre-treated with a single compound, only HVA significantly reduced the production of IL-6, while the other three phenolic acids had no effect (Fig. 4a). However, when 4 phenolic acids were applied together, we observed a synergistic effect and the level of IL-6 was significantly lower compared to the culture that was treated with HVA alone (Fig. 4a). Similarly, we found none of the phenolic acids significantly reduced TNF-α production when applied alone. 3-HPAA treatment significantly increased TNF-α (Fig. 4b). However, when the 4 phenolic acids were applied together, there was a significant reduction of TNF-α (Fig. 4b). Cell viability assessment by Trypan Blue staining showed that the cells were all viable following the treatment and following LPS stimulation.

### Treatment with FRP promotes resilience to RSDS-induced social avoidance behavior

**Fig. 5:**
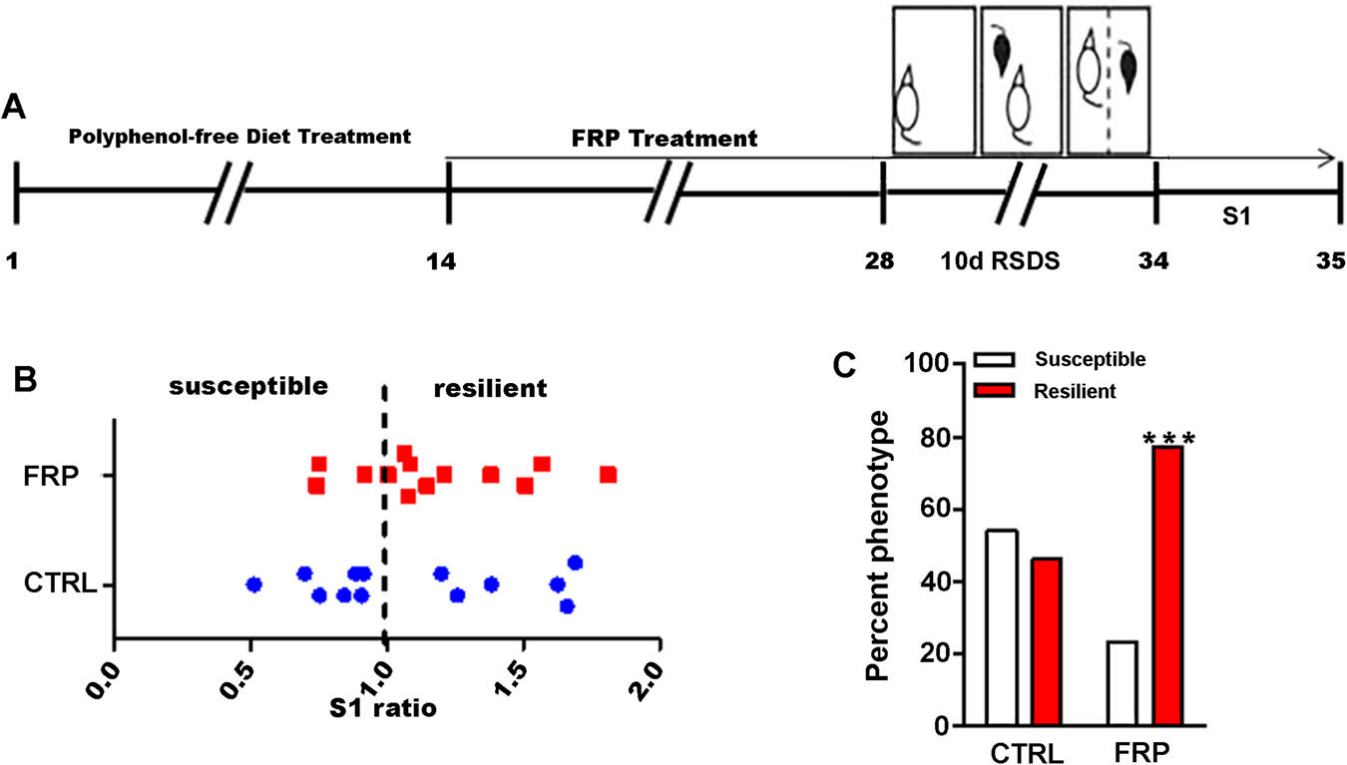
Prophylactic effect FRP in promoting resilience to social stress. **a** Schematic design of the experiment. **b** Treatment with FRP increases the proportion of mice showing a resilient phenotype, as measured by social interaction ratio (**c**) percentage of susceptible and resilient mice following FRP and vehicle treatment. Bar graphs represent mean ± s.e.m., Chi-square test, ***p < 0.001, *n* = 13 per group.

Our dose-finding studies demonstrate that 40 mg/kg-BW/day FRP can attenuate LPS-induced IL-6 production. Based upon these results, as well as in vitro evidence demonstrating that a combination of phenolic acids can attenuate LPS-induced inflammatory cytokine production, we conducted a proof of concept study to evaluate the potential role of FRP using the well-established RSDS model. Mice were fed on a polyphenol-free diet for 10 days and were then treated with FRP or vehicle for 2 weeks prior to and throughout RSDS. Following 10 days of RSDS, mice were subjected to the social avoidance test (Fig. 5a). We found that treatment with FRP greatly increased the proportion of mice resilient to the stress as indicated by normal social interactions, compared to the vehicle-treated animals. Overall, ~77% of mice receiving FRP showed resilient behavioral phenotypes, whereas only ~46% were resilient in the control groups (Fig. 5b, c).

## Discussion

MDD has become one of the leading causes of disability worldwide. Based on the data obtained from National Institute of Mental Health, it is estimated that over 15 million people experience at least one episode of major depression each year in the United States alone. Conventional anti-depressant treatments mainly target the monoaminergic system and ~50% of the patients do not fully respond to the currently available treatment, which may reflect the heterogeneity of the pathogenesis of the disorder. Since the identification of the role of inflammation in the pathophysiology of depression in a subset of hepatitis patients treated with interferon-α^39^, there is a growing interest in the field of psychoneuroimmunology to identify inflammatory molecules/cells as potential therapeutic targets for depression. Among these, IL-6 was the most consistently observed inflammatory cytokine that is closely associated with MDD^14–16^. IL-6 has emerged as a potential promising therapeutic target for MDD.

Natural products have a history of being the source for many of the active ingredients in medications, and almost half of the drugs approved since 1994 are derived or inspired from natural products^40,41^. In recent years, cocoa products, specifically cocoa polyphenols, have received growing interest due to epidemiological observation that people consuming cocoa products such as chocolate reported either improvement in mood or attenuation of negative mood^29^. The benefits of chocolate have been in part, attributed to cocoa flavanols^42,43^. Flavanols are known to be able reduce the production of pro-inflammatory molecules and to directly or indirectly impact the signaling transduction pathways associated with inflammation^31–33^. This study was designed to test the potential contribution of cocoa flavanols in modulating depression-like behavior in a well-established mouse model of depression.

The dose study we first conducted and showed that higher dose FRP treatment (200 mg/kg/day or 500 mg/kg/day) had no effect on LPS-induced IL-6 production in mice while 40 mg/kg/day FRP treatment was very effective. This begs the question: what is the difference between the low and high dose treatment? Cocoa polyphenols, including flavanols, are extensively metabolized through xenobiotic and colonic microbiome-mediated metabolism following oral consumption, resulting in the generation of phenolic metabolites - polyphenol phase II metabolites and phenolic acids. Compared to the 40 mg/kg/day and the 200 mg/kg/day treatment, we found that oral administration of FRP led to a dose-dependent accumulation of phase II polyphenol metabolites in plasma. There was also a dose-dependent accumulation of select phenolic acid metabolites in plasma. Unexpectedly, we found that the plasma contents of some of the phenolic acids, specifically 4-HCA, HVA, 3-HPAA, and VA, were lower in mice dosed with higher dose FRP.

In vitro studies showed that when applied together, these four compounds produced a synergistic effect in reducing IL-6 and TNF-α, suggesting that these phenolic acids might be partially responsible for the inhibition of IL-6 in mice challenged with LPS. This result is consistent with some of the human studies. In a randomized, controlled, double-blinded, balanced trial with 30 healthy adults designed to test the effect of cocoa flavanols on cognitive function, Scholey et al.^42^ reported that subjects who consumed 520 mg of cocoa flavanols benefited from “mental fatigue” while subjects who consumed 994 mg of cocoa flavanols reported no benefits. These observations suggest that it is very important to consider the differential metabolism of cocoa polyphenols and the generation of various bioactive phase II polyphenol metabolites and phenolic acid metabolites when determining the dosages to be tested for human studies.

In this study, we detected at least 5 polyphenol conjugate metabolites and 14 phenolic acids with measurable amounts accumulating in plasma following oral administration. Here, we only examined four phenolic acids for their synergistic effect on anti-inflammation in vitro, and our results suggest that it is possible that some phenolic metabolites may counteract the positive effect of the other phenolic metabolites and that the overall benefits might be reduced due to potential “cancellation” effects. Future studies will continue to investigate the effect of the other phenolic metabolites on anti-inflammatory bioactivities and dissect the potential mechanisms.

As a proof of concept, we demonstrated that oral administration of FRP promoted resilience against social stress. Besides the anti-inflammatory activity, cocoa polyphenols are also known for their neuroprotective bioactivities. For example, epicatechin, one of the major components of FRP, can promote neurogenesis^44^. More pertinent to our current studies, epicatechin and its phenolic metabolites are effective scavengers of reactive oxygen species and exhibit potent antioxidant properties. Cocoa extract also contains significant level of methylxanthines including theobromine and caffeine, which can act on the central nervous system mainly through adenosine receptor signaling^45^. All of these bioactivities may contribute to the benefits of FRP in attenuating the development of depression-like phenotypes in the RSDS mouse model. Future studies will further dissect the potential contributions of each of these natural products in cocoa and their underlying mechanisms in modulating depression.
